# Recent advances in the genetics of alopecia areata

**DOI:** 10.1515/medgen-2023-2004

**Published:** 2023-04-05

**Authors:** F. Buket Basmanav, Regina C. Betz

**Affiliations:** University of Bonn Institute of Human Genetics, Medical Faculty & University Hospital Bonn Venusberg Campus 1, Gebäude 13 53127 Bonn Deutschland; University of Bonn Institute of Human Genetics, Medical Faculty & University Hospital Bonn Venusberg Campus 1, Gebäude 13 53127 Bonn Deutschland

**Keywords:** Alopecia, areata-exome, sequencing-genetic, association-autoimmune-transcriptomics

## Abstract

Alopecia areata (AA) is a common autoimmune-mediated hair loss disorder in humans with an estimated lifetime risk of approximately 2 %. Episodes of hair loss usually begin with isolated hairless patches that may progress to complete hair loss over the entire body. A familial occurrence of AA is well established, with recurrence risks of about 6–8 % in first-degree relatives. AA is a multifactorial disorder involving both environmental and genetic risk factors. Previous research has identified 14 susceptibility loci, most of which implicate genes involved in the immune response. The following review presents a summary of the latest findings from genome-wide association, sequencing and gene expression studies of AA, as well as their contribution to the recent therapeutic developments.

## Introduction

Alopecia areata (AA) is a common autoimmune-mediated disorder in humans with an estimated lifetime risk of about 2 % [1]. Episodes of hair loss typically commence with the development of isolated hairless patches, which can progress centrifugally and may merge into complete hair loss over the entire head and body. Especially the unpredictable and highly variable course, with a possible regrowth of hair up to persistent and complete hairlessness is extremely stressful for many patients. In addition to the negative effects of hairlessness on self-esteem, social confidence, and sexuality [2], it is quite often that patients without scalp hair and eyebrows are often regarded by the community as cancer patients receiving chemotherapy. Considering all of these factors together, it is not surprising that affected individuals are at higher risk of developing anxiety and depression.

The common notion for AA, is that it is a multifactorial disease with both environmental and genetic factors playing a role in its aetiology. Genome-wide association studies (GWAS) on AA have reported 14 susceptibility loci supporting the polygenic nature of disease as well as its autoimmune basis [3, 4]. Next generation sequencing (NGS) studies are still in its infancy, but the first published results suggest the involvement of hair intrinsic etiological factors to also be involved in disease pathobiology [5].

## Clinics, etiology and therapy of AA

AA is a non-scarring, reversible, and circumscribed disorder of hair loss, affecting both sexes with a sudden onset that can occur at any age and a recurrent course. Of note, AA occurs with an increased frequency among certain chromosomal aneuploidies. The most consistent association, with a prevalence of up to 9 %, has been reported for Down’s syndrome [6].

Usually, the diagnosis of AA can be clearly established on the basis of patchy hair loss and the typical clinical course. Also characteristically are so-called exclamation mark hairs, short broken hairs at the edge of hairless areas. The site predominantly affected by AA is the scalp, although all hair-bearing areas of the skin may be affected. AA is divided into three main clinical types based on the extent of hair loss and the sites affected: patchy AA, AA totalis (AT) affecting the whole scalp, and AA universalis (AU) affecting all types of hair on scalp, face and whole body. AA is often associated with several other clinical features such as nail involvement, including diffuse fine pitting, longitudinal ridging, and severe nail dystrophy.

Both in the literature and from observations in our own cohort, it is known that AA patients can be comorbid for other conditions including atopy (hay fever, asthma, atopic dermatitis) and autoimmune diseases. The prevalence of different comorbidities varies widely across publications, depending on study design, ethnicity, and inclusion of different disorders. In one of the largest (almost 70.000 patients) and most recent studies, the most common comorbidities were reported to be hyperlipidemia (22.4 %), hypertension (21.8 %), thyroid disease (13 %), contact dermatitis (11 %), depression and anxiety disorders (9 % each) [7]. Asthma and atopic dermatitis were observed in about 7 % and 3 % of the patients, respectively, and further autoimmune diseases (vitiligo, rheumatoid arthritis and inflammatory bowel disease) were observed in single digits (0.8–2 %) [7]. In other studies, which also include hay fever, high numbers of up to 40–50 % are reported for the atopic group. The overlap with other autoimmune diseases is not only evident in the clinical findings, but is also reflected in the molecular genetic findings described below.

There is general agreement, that the genetic basis of AA is multifactorial with both genetic and environmental influences. GWAS delivered several disease loci containing common genetic risk variants, mostly related to immunity. Based on our recent calculations, heritability estimates for common variants lie between 30 to 44 % (unpublished data).

Despite many important advances in the field, the understanding of the etiopathogenesis of AA is still incomplete. AA is regarded as a tissue-specific autoimmune disease directed against the hair follicle [8], which is likely a consequence of the loss of the immune privilege of the hair follicle [9]. The autoimmune attack in AA is mediated by T cells [10] and a particular role is implicated for cytotoxic CD8+ cells, particularly a subset of these that is NKG2D+, regulatory T (Treg) cells as well as T helper 1 (Th 1), T helper 2 (Th 2) and T helper 17 (Th 17) cells based on i) immunohistopathological observations around AA hair follicles, ii) molecular genetic studies of AA (i. e. GWAS and gene expression studies), and iii) immune cell and cytokine phenotyping in AA patients.

The treatment of AA involves suppression or stimulation of the immune response, using treatments such as topical or intralesional corticosteroids and immunotherapy with allergic contact sensitizers, respectively [11]. Currently available therapies have low to moderate efficacies depending on the duration and extent of disease. However, based on clinical observations [12], but also on the mechanistic follow-up of gene loci identified in GWAS [4, 13], the JAK-STAT-pathway and its inhibition via JAK inhibitors came into focus as a new therapeutic approach [11]. Several clinical trials of repurposed JAK inhibitors have reported hair regrowth in ~ 60 % of patients with moderate to severe hair loss [14–18] and phase III clinical trials are ongoing. However, follow-up reports show that in the vast majority of the cases the hair fell out again after the end of therapy, suggesting that long-term treatment is necessary for maintaining hair growth [19]. Furthermore, the side effects of JAK inhibition (e. g. viral infections, thrombo-embolic events, elevated liver enzymes, serious cardiac events, lymphoma and other cancers), especially for long-term use, should not be underestimated. In addition, the high costs of JAK therapies [20] are currently not covered by health insurances in Germany, as hairlessness is not considered a disease but a cosmetic problem. Therefore, there is still a high demand for new effective and reliable treatment strategies.

## Familiarity and genetic studies in AA

Despite the multifactorial aetiology, a familial clustering of AA has been suggested by many studies. Nevertheless, there are very few studies and data on recurrence risks. In 2006, we interviewed relatives of affected persons in a German-Belgian study and calculated lifetime risks based on these interviews [21] and our findings are still of relevance for genetic counselling. First-degree relatives have a risk of 6–8 % for developing AA, second-degree relatives have a risk of 1–3.5 %, which is nearly similar to the risk of the general population. Detailed data can be found in earlier articles [21, 22]. The course and severity of the disease cannot be predicted, and both very mild and severe courses are included in this data. For AA, there are established guidelines for clinical studies to document the clinical phenotype [23, 24], which can also be used for genetic studies after appropriate adaptation. Thus, for genetic studies, a lifetime view with detailed documentation of the most severe episode, disease onset, and disease progression is more important than a description of the current episode. In addition, a detailed family history should be taken to estimate the degree of familial burden.

## Molecular genetic studies

In recent years, molecular genetic approaches to genetically complex diseases have undergone a major transformation. After candidate gene studies in the 90’s and 2000’s, which were mostly hypothesis-based, genome-wide association studies (GWAS) have come to the fore in the last decade. This is also the case in AA. We described numerous candidate gene studies in detail in our 2007 review article [22] and we will not discuss them in the current article, even though some of the identified associations are consistent with susceptibility genes that were identified by GWAS [3, 25]. In addition to the etiological insights gained from GWAS, genome-wide gene expression studies performed in human and mice models of AA also contributed substantially to the understanding of disease pathobiology and delivered molecular signatures of disease state [11].

### Common and rare risk variants in AA

Despite the large number of affected individuals worldwide, to date only two systematic GWAS have been performed for AA with smaller collectives [3, 4] compared to other genetically complex phenotypes. The majority of the 14 loci obtained are related to immune responses and the strongest region of association is the major histocompatibility complex (MHC) [3, 4]. In more detail, the first GWAS including 1.000 patients from a national registry from North America resulted in the identification of eight genomic regions that had been significantly associated with AA [4]. The majority of these loci mapped to genes involved in innate and adaptive immunological responses including the HLA region (top association signal), and the genes *IL–2/IL–21*, *IL–2RA*, *CTLA4*, *IKZF4* and *ULBP3/ULBP6*. Two loci indicated genes expressed in the hair follicle, namely, *STX17* suggesting a role for end-organ autophagy, and *PRDX5* implicating oxidative stress [4]. Interestingly, a large number of the implicated GWAS genes including *IL–2/IL–21*, *IL–2RA*, *CTLA4*, and *IKZF4* are primarily involved in regulating proliferation, differentiation and activity of T regulatory (Treg) cells. The *IL–21* locus, on the other hand, also implicates a role for proinflammatory Th17 cells, as IL–21 is a product of the Th17 cells promoting their differentiation while limiting the differentiation of the antagonist Treg cells [26]. The second strongest association signal emerged from the locus mapping to *ULBP3* and* ULBP6* genes [4] encoding for ligands of the NKG2D receptors [27] expressed on a subset of CD8+ T-cells, natural killer cells, natural killer T cells and δγ T cells. This finding together with immunohistochemistry analyses provided a very important etiological insight into AA: *ULBP3* is upregulated in the hair follicles of AA patients and may act as an inducer of NKG2D-mediated immune response against the hair follicle [4, 28, 29].

In a subsequent follow-up study, we analysed the SNPs emerging from the first AA GWAS in our own cohort of European (EU) origin and replicated the previous associations from five of the established loci [30]. Furthermore, a joint analysis of our genotype data and the US data allowed two of the previously nominally significant loci, namely, *IL13/IL4* and *KIAA0350/CLEC16A*, to exceed the genome-wide significance threshold [30]. The second AA-GWAS combines in a meta-analysis the US and EU cohorts comprising a total of about 3.000 cases and 7.500 controls [3]. In this meta-analysis, the previously identified loci could be confirmed; and two other loci, namely *ACOXL/BCL2L* and *GARP*, surpassed the genome-wide significance threshold. *GARP* had earlier been reported in GWAS of other autoimmune and inflammatory diseases, and is expressed on activated Treg cells being responsible for the induction of *FOXP3* expression and Treg differentiation [31, 32]. In this meta-analysis, a fine mapping of the association signal from the HLA region was performed with the use of computational methods. Here, we discovered four independent effects, all of which implicate HLA-DR as the primary risk factor for AA (most significant variant with the p-value: 4.99 × 10^–73^) [3]. Finally, we also performed a cross-phenotype meta-analysis by integrating our results with data from other autoimmune diseases and identified functional clusters of genes and proteins indicating mechanistic alignment of AA with rheumatoid arthritis (RA), celiac disease, type I diabetes, multiple sclerosis and Crohn’s disease [3].

To date only a few genetic studies have addressed the contribution of rare variants to the risk of developing AA. A previous linkage analysis in families with multiple affected individuals had identified several genetic regions that co-segregated with the disease phenotype [33]. This study had not identified any individual genes or variants, and it seems that linkage analysis approach in familial AA has not been further pursued in the era of next generation sequencing (NGS). To date, three NGS studies were reported in AA. Two of these are underpowered studies, each with 6 and 50 patients [34, 35], respectively, and the third one is a recently published WES study from the US cohort which presents the analysis of data from 849 AA patients and 15.640 controls [5]. The authors identified a higher burden of rare, damaging *KRT82* variants in patients in comparison to healthy individuals at a genome-wide significance level. In more detail, they found deleterious mutations in *KRT82* in 6 % of the AA patients included in the study in comparison to 2.58 % of the controls. Most interestingly, the authors showed a decreased expression of KRT82 in skin and hair follicles of patients with AA [5]. *KRT82* encodes for a type-II keratin exclusively expressed in the hair shaft cuticle and together with other hair keratins, it plays a role in the formation of intermediate filaments necessary for the structural integrity of the hair shaft [36]. Interestingly, mutations in other type-II keratins are associated with monogenic hair disorders, e. g. monilethrix [37]. Aberrant expression of *KRT82* and its consequences on the hair shaft integrity may be involved in the pathophysiology of AA with further mechanistic insights awaiting identification.

In addition to these rare single nucleotide variants identified in *KRT82* by WES, using genome-wide SNP array data we and others have provided some evidence that large, rare, structural variants, namely, copy number variants (CNVs) may also play a role in the etiology of AA. Petukhova et al. [38] identified in the US cohort a significantly higher genome-wide burden of rare, large, gene-disrupting CNVs in AA patients in comparison to healthy controls. The US group identified 14 genes whose expression levels were altered by CNVs in a consistent direction of effect, corresponding to gene expression changes in lesional skin of AA patients. Four of these genes were affected by CNVs in three or more unrelated AA patients, including the genes *ATG4B* and *SMARCA2*, which are involved in autophagy and chromatin remodeling, respectively [38]. Our group also performed a genome-wide- and a candidate gene-focused CNV analysis in 585 patients and 1.340 controls. We identified nominally significant associations of CNVs with AA in five chromosomal regions. The most promising finding was a 342.5-kb region in 6q16.3 (duplications in 4/585 patients; 0/1.340 controls; *P*=.008) [39]. The duplications spanned the genes *MCHR2* and *MCHR2-AS1*, implicated in melanin-concentrating hormone (MCH) signaling [40]. AA preferentially affects pigmented hairs, and the hair of patients with AA frequently shows a change in color when it regrows following an acute episode of AA. Together with our findings this may indicate a relationship between pigmentation, MCH signaling and AA.

The application of other sequencing technologies including whole genome and long read sequencing will deliver further insights into the role of rare and structural variants in AA and studies employing these approaches are expected to emerge in the near future.

### Genome-wide gene expression studies in AA

To date, several genome-wide gene expression studies have been performed using scalp skin biopsies from AA patients as well as skin tissue from murine models of AA [13, 41–46]. In general, genome-wide gene expression studies in AA generated converging and consistent evidence for 1) upregulation of interferon gamma (IFN-γ) response genes, (e. g. *CXCL9*, *CXCL10*, and *CXCL11,* encoding for inflammatory chemokines) and 2) downregulation of hair keratin (KRT) and keratin associated protein (KRTAP) genes in AA. IFN-γ is a major cytokine of the Th 1 mediated immune response. Today, the Th 1 immune axis (and particularly IFN-γ) is widely accepted as one of the main drivers of AA pathogenesis based on several lines of evidence one of which is the consistent identification of gene expression signatures related to IFN-γ signaling by gene expression studies. Other lines of evidence come from immunohistological observations showing the presence of IFN-γ producing immune cell infiltrates around the AA hair follicles, AA GWAS loci and cytokine/immune cell phenotyping studies in AA patients.

Besides providing important insights into disease pathobiology, genome-wide gene expression studies contributed to the consideration of novel treatment approaches in AA. The first genome-wide gene expression study in AA was performed in mice and humans in 2002 and suggested that *CTLA-4* expression is dysregulated in AA [45], a finding that was later corroborated by GWAS which identified *CTLA-4* as a susceptibility locus [3, 4]. These discoveries served as a rationale for the consideration of repurposing of abatacept, a CTLA-4 mimetic [47], for the treatment of AA [48]. The recently reported results of this clinical trial (NCT02018042) suggested an efficacy that can be considered as low to moderate [49].

The most important data-driven drug repurposing attempt in connection with transcriptome data is based on a milestone study that was published in 2014 [13]. In this study transcriptional profiling of mouse and human AA skin demonstrated significant dysregulation of i) cytotoxic T lymphocyte (CTL) transcripts, ii) IFN response genes and iii) genes encoding for gamma chain (γC) cytokines IL–2 (also a GWAS locus) and IL–15 and their receptors, which are known to promote the activation and survival of IFN-γ-producing CD8+ NKG2D+ T cells. IFN-γ receptors and γC family receptors signal through JAK1/2 and JAK1/3 kinases, respectively and therefore, this discovery led the authors to consider interfering with these downstream effectors as a therapeutic modality in AA. Accordingly, they treated AA mice and three human subjects with two small molecule JAK inhibitors, which were FDA-approved for the treatment of other diseases. These patients represented to be the first treated participants of the first open label proof-of-concept clinical trial of JAK inhibition initiated by the authors (NTC01950780) where they showed the high efficacy of JAK inhibition in promoting hair regrowth. This observation, together with independent case reports of unexpected hair growth in patients treated with JAK inhibitors against other autoimmune conditions with concomitant AA [12] led to the further pursue of larger clinical trials of JAK inhibition in AA.

Despite the common clinical observation that AA often co-manifests with atopic diseases, the role of Th 2 mediated immune response (traditionally related to atopy) was controversial in AA for quite a long time. First, genetic association studies delivered the *IL13/IL4* locus encoding for cytokines of Th 2 immune response [3, 30] and then a large genome-wide gene expression study performed using scalp skin biopsies of 27 AA patients and 6 controls in 2015, showed that genes involved in Th 2 immune response are also dysregulated in AA patients [41]. These findings landed an important support to the involvement of Th 2 immune axis in the pathogenesis of AA and most importantly, contributed (together with other lines of evidence [50–55]) to the consideration of Th 2 antagonism as a novel therapeutic modality in AA. The recently published results of a clinical trial of dupilumab (anti-IL–4Rα, a monoclonal antibody inhibiting Th 2 signaling and an FDA approved drug for atopic dermatitis) in AA, revealed promising results with what may be considered as a moderate efficacy, particularly in individuals with a baseline IgE level ≥ 200 IU/ml and a personal or family history of atopic disease [56]. Of note, findings from the transcriptome study in 2015 [41] led to consideration of two other repurposing based therapeutic approaches (i. e. ustekinumab and apremilast), however, in each of these, the therapeutic effect was limited or controversial [11, 57, 58].

Besides inspiring drug repurposing attempts, identification of consistent patterns of gene dysregulation in AA led previous authors to conceive the so called gene expression signatures: Alopecia Areata Disease Activity Index (ALADIN) [13, 42] and Alopecia Areata Gene Signature (AAGS) [46]. ALADIN is a three-dimensional quantitative composite gene expression score, composed of 16 genes belonging to one of the three gene clusters, namely IFN response genes, CTL genes and KRT genes. AAGS on the other hand is composed of 136 genes with 123 of these belonging to skin-specific molecular programs and the rest to the immune tissue. The possible use of these gene signatures for clinical purposes was evaluated by several studies. For example, ALADIN was suggested to enable a quantitative assessment of disease severity or status in AA and distinguish severe AA (AT/AU) from mild AA (patchy AA) [13, 42]. Furthermore, as we have reviewed in detail elsewhere [11], analyses of pre- and post-treatment transcriptome data in several clinical drug trials have shown that such gene expression signatures i) can be used to monitor the patients’ response to drugs on a molecular level [59–62] and ii) may hold the potential to serve as predictive biomarkers of drug response [61–63]. However, the clinical utility of such findings should be validated and evaluated by prospective and longitudinal studies in large cohorts.

## Summary and outlook

AA cohorts genetically analyzed to date and accordingly the number of susceptibility loci they delivered are relatively modest in comparison to other complex diseases, some of which have delivered up to hundreds of loci using collectives of several 100.000 affected individuals. Therefore, it is definitely warranted to perform further genomics research in AA by i) assembling larger cohorts and/or ii) applying NGS approaches to identify novel common and rare variant associations which may have important implications for molecular diagnostics and can point towards novel drug targets [11, 64]. Current therapies are based on the suppression of the immune response on a general basis, which is associated with important side effects. The discovery of novel loci may point towards skin/hair follicle related pathways which may serve as more specific drug targets associated with less systemic side effects. In this line, aiming to maintain or restore the hair follicle immune privilege as a novel therapeutic concept can prevent relapses and have a revolutionary impact in the management of AA [65, 66].

Genome-wide gene expression studies in AA delivered consistent patterns of gene dysregulation in AA, which contributed to the understanding of disease pathogenesis and consideration of novel therapeutic approaches. Furthermore, gene expression signatures derived from these studies were suggested to be useful for monitoring patients’ response to drug treatment on a molecular level and may serve as predictive biomarkers of drug response [11]. Therefore, the further pursue of transcriptional profiling studies in AA also holds important potential. However, obtaining scalp skin biopsies, is an important limitation and it is unrealistic to expect that large samples will be collected. Here, opting for single-cell RNA sequencing (RNA-seq) approach by using samples from a few subjects can be even more beneficial, particularly considering the extensive complexity of the ecosystem of skin immunity and diversity of cell types involved in the pathogenesis of AA. Single-cell RNA-seq provides a high resolution enabling the generation of novel insights into pathogenic mechanisms operating in distinct cell populations, which may prime the development of novel therapies on the long run. To date, only one single-cell RNA-seq study was performed in AA, which analyzed the expression profiles of immune cells of a murine model of AA and T-lymphocytes of a human subject [67]. The authors identified differences in the antigen presenting cells and T cells of unaffected skin and AA skin in the murine, which were predictive of human disease state. More single-cell RNA-seq studies are expected to emerge in the near future, as the use of this technology will become more conventional in the upcoming years.

The last but not least, in our opinion, one of the most important prospect for future molecular genetic studies is to detangle or deconstruct the clinical heterogeneity observed in AA (in terms of disease course, concomitant presentation of atopy/other autoimmune diseases and therapy response) on the molecular level [11]. The identification of genetic and transcriptional biomarkers correlated with these clinical entities can be achieved by the application of -omics technologies in phenotypically well-characterized patient cohorts. This emphasizes the importance of integrating accurate phenotyping tools to the patient recruitment efforts.

To conclude, both GWAS and genome-wide gene expression studies have delivered important insights into the etiology and pathobiology of AA and inspired some translational research. However, the efficacies of the conventional and repurposing-based therapeutic modalities are still limited and JAK inhibitors with better efficacies necessitate long-term therapy, are associated with severe side effects and are not accessible to the vast majority of the patients. Therefore, further pursue of molecular genetic studies is warranted to point novel drug targets and identify biomarkers for developing personalized medicine tools.
